# Electrocautery vs. Stapler in Comparing Safety for Segmentectomy of Lung Cancer: A Meta-Analysis

**DOI:** 10.3389/fsurg.2021.711685

**Published:** 2021-08-04

**Authors:** Tianjian Lu, Ruoxi Zhang, Kexin Jiang, Zihuai Wang, Xiaohu Hao, Nan Chen, Lunxu Liu

**Affiliations:** ^1^Department of Thoracic Surgery, West China Hospital, Sichuan University, Chengdu, China; ^2^West China School of Medicine, Sichuan University, Chengdu, China; ^3^Western China Collaborative Innovation Center for Early Diagnosis and Multidisciplinary Therapy of Lung Cancer, Sichuan University, Chengdu, China

**Keywords:** lung cancer, segmentectomy, postoperative complication, electrocautery, lung

## Abstract

**Background:** Electrocautery and staplers are regarded as the two most common surgical instruments for dissecting the intersegmental plane in segmentectomy. We performed a meta-analysis to compare electrocautery and staplers in terms of their safety and effects.

**Methods:** A systematic search strategy was performed using PubMed, and the retrieval time was up to April 1, 2020. Odds ratio (OR) and mean differences (MDs) with 95% CI were applied to determine the effectiveness of dichotomous or continuous variables, respectively.

**Results:** Six studies including 385 patients were included. The electrocautery had a higher incidence rate of postoperative complication [OR= 1.92, 95% CI (1.12, 3.28), *P* = 0.02)] and air leak [OR: 3.91, 95% CI (1.64, 9.35), *P* = 0.002)]. No significant difference was found in the comparison of surgery time, blood loss, and duration of tube days or hospitality days.

**Conclusions:** Our study indicated that patients under segmentectomy were associated with better safety by using stapler than electrocautery in the reduction of postoperative complications.

## Introduction

Lung cancer has been primary cancer with the highest incidence rate around the world. It is the leading cause of cancer-related mortality. Based on the eighth National Comprehensive Cancer Network (NCCN) guidelines of non-small cell lung cancer, segmentectomy is one of the most frequently used radical operations for early-stage lung cancer (1, 2). This procedure applies to the early-stage or “limited” primary tumor that does not invade into the 1 cm line to the border of the located segment. Segmentectomy has the advantages of preservation of pulmonary function (3), a shorter hospital stay, and fewer postoperative complications over lobectomy (4–6). This operation has also been confirmed safe (7, 8). The recognition and division of intersegmental planes are the key steps in segmentectomy (9). Two surgical instruments are recommended for the approach to intersegmental planes, namely, electrocautery and staplers (2). However, further investigations are required to compare these two instruments. In this study, we performed a review and a meta-analysis to compare the short-term safety of the intersegmental plane division between electrocautery and staplers.

## Methods

### Literature Review and Data Extraction

A systematic literature review was performed on April 1, 2020, using the search term [“intersegmental” (Mesh)] plane in PubMed. All reported pulmonary segmentectomy (including open surgery and video-assisted thoracoscopic surgery) for primary lung cancer was screened. Prospective and retrospective studies comparing the usage of electrocautery and stapler in segmentectomy were included in this meta-analysis. The information of surgery performed under either open surgery or minimally invasive surgery was included, and patients were divided into two groups based on the surgical instruments for separating the levels between segments. The electrocautery group adopted electrocautery as the main approach, while the stapler group mainly used the staplers, with or without using electrocautery as the assistant method. Besides, studies written in English or Chinese were included as long as the data were recorded in English and can be completely approached. The exclusion criteria included the following: (1) Complete data of operative information of patients were not approached, (2) studies with only one group of one instrument and no comparison, (3) comparison with other surgical instruments instead of electrocautery and staplers, (4) no sublobular resection or segmentectomy for the surgical method of lung cancer, and (5) the types of articles that were not available, such as abstracts, reviews, letters, book chapters, animal experiments, and case reports.

Two researchers independently extracted the number of cases, age and gender of patients, and outcomes including operation time, tube duration, the number of major postoperative complications (including atelectasis, hemorrhage, pneumonia, and pulmonary embolism), and the number of air leaks (lasting more than 7 days). If not explicitly quoted, mean differences (MDs) and *p*-values (based on *t*-tests) were used to express SEs.

The Newcastle-Ottawa (NOS) scale was used to evaluate the quality of retrospective studies; those scoring 6 or higher were considered qualified. The quality of prospective cohort studies and randomized controlled trials (RCTs) was assessed by the Jadad scale (10), and those scoring 4 to 7 were deemed as high-quality studies.

### Statistical Analysis

We used Review Manager V5.3 (The Cochrane Collaboration, Software Update, Oxford, UK) for extraction, pooling, and analysis of data, and assessment of the risk of bias (tool of The Cochrane Collaboration). Odds ratios (ORs) and corresponding 95% CIs were used together to evaluate the dichotomous outcomes. The curative effect in continuous variables was expressed as MDs with corresponding 95% CI. *P*-values lesser than 0.05 were identified as statistically significant. Statistical heterogeneity was assessed by *I*^2^ statistics. In particular, *I*^2^ < 50% represents no statistically significant heterogeneity across studies, and the fixed-effects model should be used. However, if *I*^2^ > 50%, the randomized-effects model would be applied. Each study was sequentially removed in the sensitivity analysis.

## Results

### Study Characteristics

**Figure 1:**
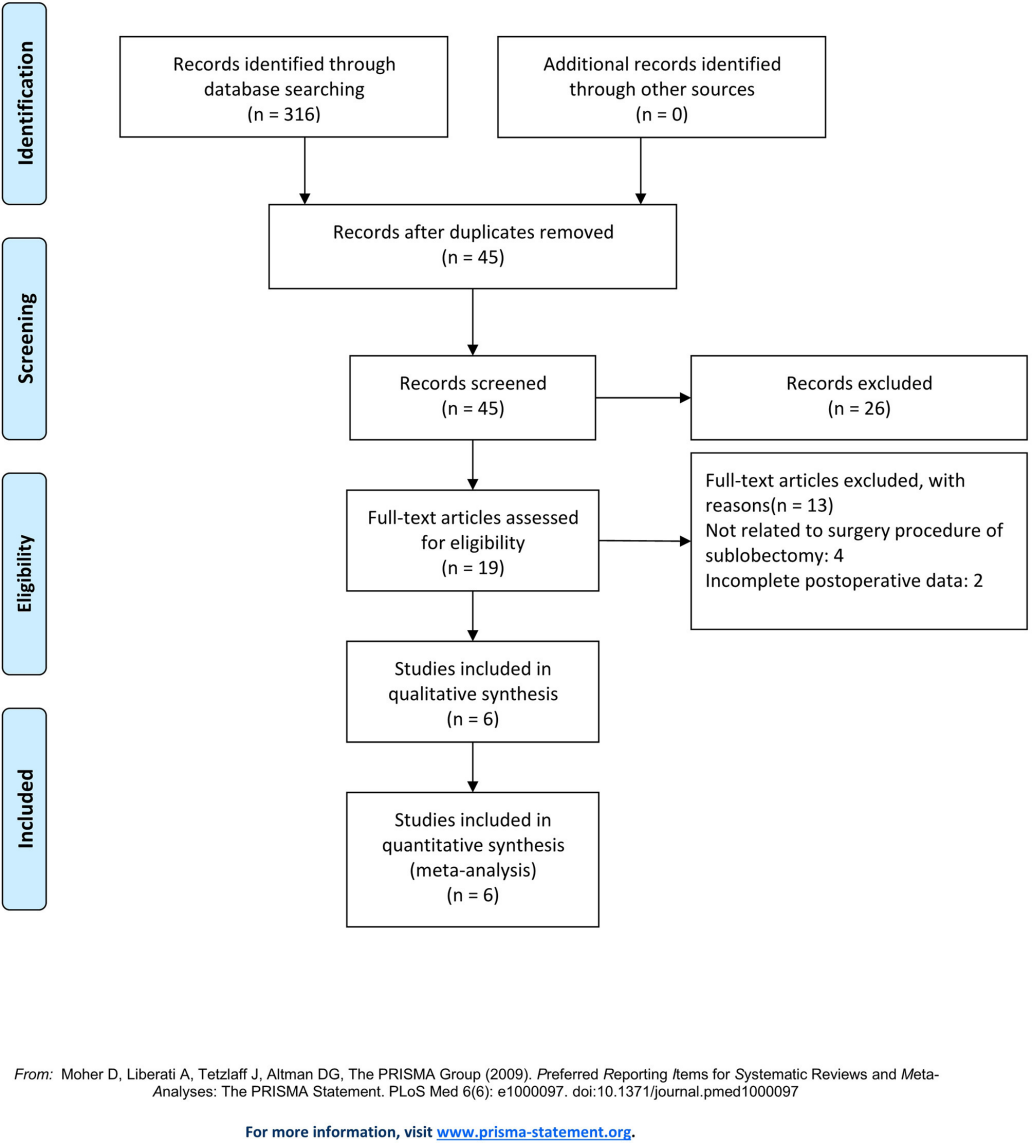
Flowchart of the study search and selection.

A total of 316 records were identified in PubMed. After a systematic review of the abstract in each publication, 297 studies were excluded, and the full text of the other 19 studies was reviewed. After evaluation, six studies were eventually enrolled (11–16), including five retrospective observational studies and one RCT, with a total of 385 patients. The single-arm research studies and studies with no description of the comparison of surgical instruments were excluded. Studies included are listed in Table 1. The evaluation procedure is shown in Figure 1.

**Table 1:** Characteristics of the included clinical trials.

**Study**	**Year**	**Country**	**Study design**	**Gender(M/F)**	**Sample size E/S**	**Propensity score maching**	**Quality assessment**
Miyasaka (11)	2010	Japan	Retrospective	21/28	31/18	Unmatched	NOS:7
Othuka (12)	2012	Japan	Retrospective	22/25	22/25	Unmatched	NOS:7
Tao (13)	2016	Japan	Retrospective	19/22	22/19	Unmatched	NOS:8
Liu (14)	2017	China	Retrospective	25/33	30/28	Unmatched	NOS:8
Matsumoto (15)	2018	Japan	Retrospective	72/53	50/75	Unmatched	NOS:7
Chen (16)	2020	China	RCT	24/41	32/33	matched	Jadad:6

### Study Characteristics and Assessment of the Risk of Bias

All studies were published before April 2020. Of all the patients involved, 187 (48.6%) underwent electrocautery-based segmentectomy and 198 (51.4%) received stapler-based segmentectomy. Table 1 shows the characteristics and details of included studies. The Jadad and NOS scores were used to assess the quality of the RCT and retrospective observational studies. No study was evaluated as low quality. Details of NOS results of retrospective observational studies are available in Table 2.

**Table 2:** Risk of bias assessment of included cohort studies.

**Study**	**Selection**				**Comparability**	**Outcome**			**Total score**
	**Exposed cohort**	**Ascertainment of exposure**	**Outcome of interest**	**Non-exposed cohort**		**Assessment of outcome**	**Length of follow-up**	**Adequacy of follow up**	
Miyasaka	^*^	^*^	^*^	^*^	–	^*^	^*^	^*^	7
Othsuka	^*^	–	^*^	^*^	^*^	^*^	^*^	^*^	7
Tao	^*^	^*^	^*^	^*^	^*^	^*^	^*^	^*^	8
Liu	^*^	^*^	^*^	^*^	^*^	^*^	^*^	^*^	8
Matsumoto	^*^	^*^	^*^	^*^	–	^*^	^*^	^*^	7

* *Means 1 score for the included study in NOS system*.

### Primary Outcome Measures

**Figure 2:**
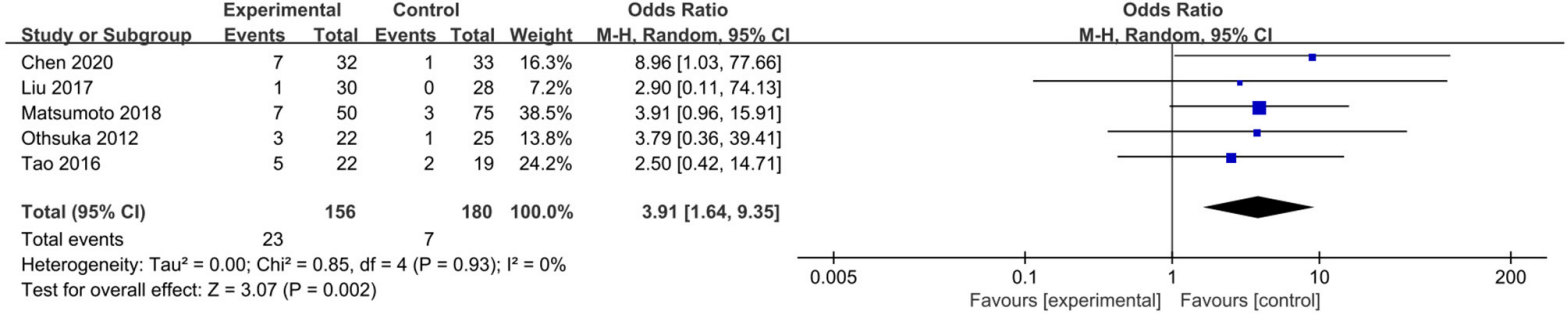
Forest plot of air leak rate comparing using electrocautery to stapler.

The electrocautery group was taken as the experimental group, and the stapler group was regarded as the control group in our analysis. Five studies focusing on the incidence of air leaks presented that there were 23 in 156 patients (14.7%) suffering from air leaks in the electrocautery group and 7 in 180 patients (3.9%) suffering from air leaks in the stapler group (12–16). The incidence of postoperative air leaks in the electrocautery group was higher than that in the stapler group [OR: 3.91, 95% CI (1.64, 9.35), *P* = 0.002] (Figure 2). No heterogeneity (*I*^2^ = 0, *P* = 0.93) or publication bias was observed.

**Figure 3:**
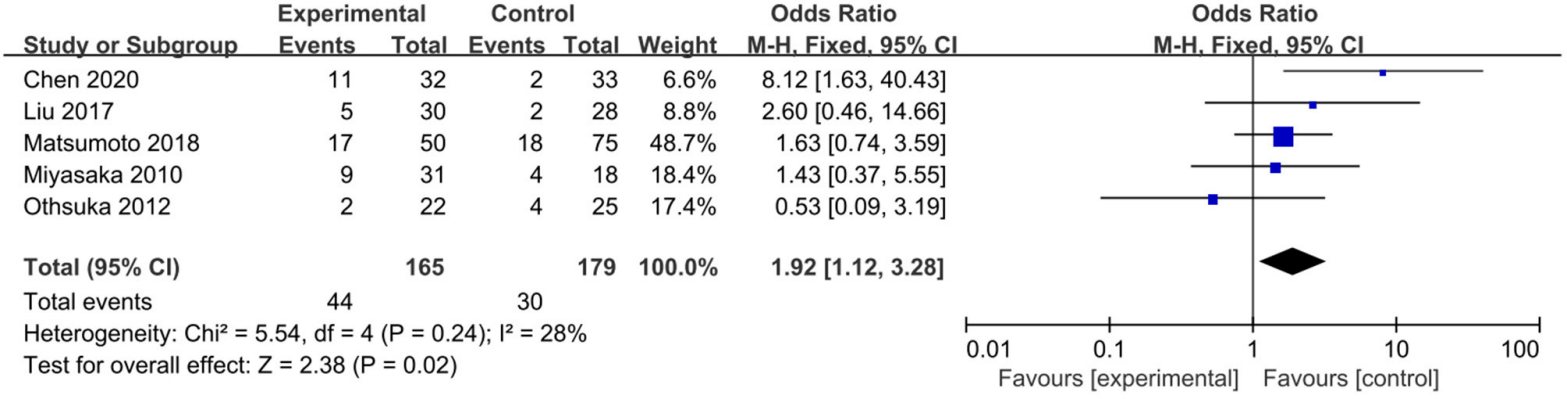
Forest plot of the overall rate of postoperative complications comparing using electrocautery to stapler.

**Figure 4:**
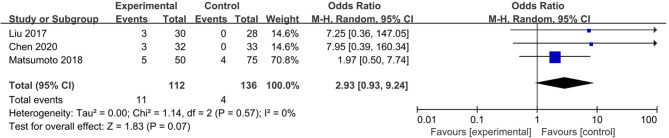
Forest plot of rate of postoperative pulmonary complications comparing using electrocautery to stapler.

Postoperative complications indicated the safety of the operation. Five studies revealed that 44 in 165 patients (26.7%) in the electrocautery group and 30 in 179 patients (16.8%) in the stapler group developed postoperative complications (11, 12, 14–16). The incidence of complications in the electrocautery group was significantly higher than that in the stapler group [OR: 1.92, 95% CI (1.12, 3.28), *P* = 0.02] (Figure 3). The result was credible with low heterogeneity (*I*^2^ = 28%, *P* = 0.24). Among these five studies, three reported respiratory complications: 11 in 112 patients (9.82%) of the electrocautery group and 4 in 136 patients (2.94%) of the stapler group (14–16). The incidence of respiratory complications (including pneumothorax, chylothorax, and atelectasis) in the electrocautery group was significantly higher than that in the stapler group [OR: 2.93, 95% CI (0.93, 9.24), *P* = 0.07]. The result was stable with no heterogeneity (*I*^2^ = 0%, *P* = 0.57). Detailed information is presented in Figure 4.

### Secondary Outcome Measures

The surgery time was compared in four studies (12, 14–16) including 295 patients, and there was no significant difference between the two groups [MD = 18.07, 95% CI (−10.89, 47.04), *P* = 0.22]. The result was confirmative with high heterogeneity (*I*^2^ = 77%, *P* = 0.004).

Four studies (12, 13, 15, 16) including 288 patients focused on the duration of the drainage tube and showed no significant difference between the two groups [MD = 0.36, 95% CI (−0.27, 0.98), *P* = 0.26]. The details of the above and other outcomes are summarized in Table 3.

**Table 3:** Secondary outcome measurements.

**Outcomes**	**Studies included**	**Pooling model**	**Effect size**	**95%CI**		***P***	***I*^**2**^**
Surgery time	4	Random	18.07	−10.89	47.04	0.22	77%
Duration tube days	4	Fixed	0.36	−0.27	0.98	0.26	0
Hospitality days	2	Fixed	0.58	−0.41	1.57	0.25	0
Intraoperative blood loss	2	Fixed	0.18	−67.75	68.11	1	45%

### Sensitivity and Publication Bias

Sensitivity analysis was applied by sequentially removing all included studies one by one to find the source of heterogeneity. The results were stable among all the included studies. Publication bias was assessed by Review Manager V5.3. Limited by the number of eligible studies, no funnel plot was drawn for the evaluation of data.

## Discussion

It remains a controversy whether segmentectomy is a safe and effective surgical treatment approach for lung cancer. According to several recent research studies, segmentectomy (including open surgery and minimally invasive surgery procedures) is now a conventional therapy for early-stage lung cancer (7, 17–19). Although only one high-quality RCT focusing on the relationship between the safety and the usage of surgical instruments was enrolled in this study, it is the first systematic review and meta-analysis designed to assess the effects and safety of electrocautery and staplers used in segmentectomy.

The most appropriate surgical instruments for segmentectomy in patients with lung cancer have been debated for a long time (20, 21). It is generally believed that electrocautery is more applicable to primary tumor removal because its smaller and more flexible front end enables the precise outlining of the tumor boundaries and thus apparently reduces the removal of normal lung tissues and reserves more pulmonary functions for patients. Besides, electrocautery may be more effective in a resecting primary tumor located in some particular pulmonary segments. However, the thermal separation in electrocautery depends on electric energy, which is more likely to cause heat damage of the incisal edge of normal lung tissues. Moreover, the lung tissues excised by electrocautery are difficult to bond automatically, which possibly leads to postoperative complications. A variety of covering materials has been used to protect the surgical section; for instance, Droghetti et al. (22) performed pleurodesis with autologous pleurodesis for treatment of postoperative persistent air leaks, which indicated an optimistic outcome of safety and efficiency. However, the procedures using staplers are more likely to preserve the section automatically and to prevent the occurrence of complications (23). A retrospective study performed by Tao et al. (24) in 2019 proved that the loss of pulmonary function caused by using staplers was acceptable. Given the unclear result, most surgeons are inclined to choose the instruments by individual habits (25). Electrocautery has other merits such as preserving more pulmonary functions and a lower cost (14). However, Chen et al. (16) found no significant advantage of electrocautery in terms of the efficiency and safety in their RCT. Besides, the convenience of using stapler in the ligation of the segmental blood vessel and bronchus is also an advantage of stapler than electrocautery. Thus, when to use electrocautery in segmentectomy is still questionable.

Our meta-analysis indicated that the stapler might be superior to electrocautery since it showed fewer postoperative complications and air leaks. Besides, there was no distinctive difference in surgery time, tube duration, and the hospital stay between patients using electrocautery and staplers. Above all, the results tended to prove that the stapler was safer in surgical procedures, which was consistent with the findings of the RCT (16). However, the secondary conclusion should be interpreted with caution due to the limited number and quality of enrolled trials. Though a higher rate of postoperative complications (including air leaks) was found in the electrocautery group, it seemed to have no influence on postoperative recovery in the two groups. This result may be associated with a limited rate of air leaks and other complications. Overall, in our submission, using staplers in segmentectomy seems to be a better choice to reduce the incidence of complications. However, there still remains valuable attention for delayed air leak in segmentectomy. A former RCT (26) compared the rate of postoperative air leak in segmentectomy between using stapler and electrocautery. The outcomes showed a lower air leak time (1.7 vs. 4.5 days) and less cost (425 vs. 630.5 euros) in the electrocautery group accompanied by the usage of human fibrinogen and thrombin than stapler group for the completion of interlobar fissures during pulmonary lobectomy, which may indicate another proper range for the usage of electrocautery. Besides, the usage of other surgical techniques is also under controversy, such as the usage of three-dimensional reconstruction and Harmonic ultrasonic scalpel (21).

Our study has some limitations. First, we included five retrospective studies and one RCT but no eligible prospective study, which may lower the quality of data. Second, it was difficult to stratify potential confounders such as age and the stage of cancer, which, however, are closely associated with clinical complications. Third, part of our study included a surgical strategy that applies one of the surgical instruments mainly but still uses another instrument, which may lead to confounding bias. Fourth, because of the differences between the attentions of outcome measurements in each study, the number of patients evaluated for each outcome is lower than the total number of those enrolled in our study, especially for the secondary outcomes, which may limit the quality of the conclusion of our study. Finally, the impact of the procedure on long-term prognosis was yet unclear. Chen et al. attempted to observe the long-term survival outcome but failed due to a marked difference in the short-term outcome. More original research studies are warranted to evaluate the influence of segmentectomy and staplers on the survival of the patients.

## Conclusions

In conclusion, our systematic review and meta-analysis indicate that staplers are safer than electrocautery and can reduce the incidence of air leaks and postoperative complications. Besides, no significant difference is found in some other primary postoperative indexes (including tube duration and surgery time) between patients using segmentectomy and staplers. Due to the limitations of included research studies, more RCTs and prospective studies of high quality are expected to reach further conclusions on the proper surgical instruments.

## Data Availability Statement

The original contributions presented in the study are included in the article/supplementary materials, further inquiries can be directed to the corresponding authors.

## Author Contributions

LL, TL, and RZ designed the study. RZ and TL collected the data and analyzed the data. TL, RZ, KJ, and ZW wrote the manuscript. LL, TL, and NC reviewed the manuscript. All authors contributed to the article and approved the submitted version.

## Conflict of Interest

The authors declare that the research was conducted in the absence of any commercial or financial relationships that could be construed as a potential conflict of interest.

## Publisher's Note

All claims expressed in this article are solely those of the authors and do not necessarily represent those of their affiliated organizations, or those of the publisher, the editors and the reviewers. Any product that may be evaluated in this article, or claim that may be made by its manufacturer, is not guaranteed or endorsed by the publisher.

## Abbreviations

95% CI: 95% confidence intervals
MD: mean difference
NSCLC: non-small cell lung cancer
OR: odds ratios
RCT: randomized controlled trial
NOS: Newcastle-Ottawa scale.
